# Interplay Between Oral Health and Cardiovascular Risk Factors in Tooth Loss: Evidence from a Cross-Sectional Analysis

**DOI:** 10.3390/life16101717

**Published:** 2026-10-14

**Authors:** Alexandra Cinezan, Camelia Bianca Rus, Luminita Ligia Vaida, Timea Claudia Ghitea, Madalina Ioana Moisi, Corina Cinezan

**Affiliations:** 1 Faculty of Dental Medicine, University of Medicine and Pharmacy, 400012 Cluj-Napoca, Romania; 2 Department of Medical Disciplines, Faculty of Medicine and Pharmacy, University of Oradea, 410073 Oradea, Romania; 3 Clinical County Emergency Hospital Bihor, 410169 Oradea, Romania; 4 Doctoral School of Biological and Biomedical Sciences, University of Oradea, 410087 Oradea, Romania; 5 Department of Dental Medicine, Faculty of Medicine and Pharmacy, University of Oradea, 410068 Oradea, Romania; 6 Pharmacy Department, Faculty of Medicine and Pharmacy, University of Oradea, 1 University Street, 410087 Oradea, Romania; 7 Department of Preclinical Discipline, Faculty of Medicine and Pharmacy, University of Oradea, 410068 Oradea, Romania

**Keywords:** tooth loss, oral hygiene, cardiovascular risk factors, smoking, diabetes mellitus, ambulatory patients, chronic inflammation

## Abstract

Background: Tooth loss is a cumulative oral-health outcome associated with reduced quality of life and several behavioral and systemic factors. Fewer studies have examined oral hygiene and multiple cardiovascular risk factors simultaneously in relation to the number of missing teeth within the same ambulatory clinical population. Our study examined the cross-sectional associations of oral hygiene status and major cardiovascular risk factors with the number of missing natural teeth in an ambulatory adult population. Methods: In this cross-sectional study, 1031 ambulatory adults underwent clinical assessment of missing natural teeth and oral hygiene status, categorized as good, fair, or poor. Hypertension, obesity, dyslipidemia, current smoking, and diabetes mellitus were recorded. Multivariable negative binomial regression adjusted simultaneously for oral hygiene status, age, sex, and the measured cardiovascular risk factors was the primary analysis, with multivariable linear regression used as a supportive analysis. Results: The mean number of missing teeth increased across oral hygiene categories. In the adjusted negative binomial model, fair oral hygiene (IRR 1.179, 95% CI 1.114–1.248) and poor oral hygiene (IRR 1.490, 95% CI 1.406–1.579) were associated with a higher expected number of missing teeth compared with good oral hygiene. Increasing age (IRR 1.031 per year, 95% CI 1.029–1.033), current smoking (IRR 1.147, 95% CI 1.099–1.198), and diabetes mellitus (IRR 1.118, 95% CI 1.056–1.182) were also associated with a higher expected number of missing teeth. Sex, hypertension, obesity, and dyslipidemia were not statistically significant in the fully adjusted model. Conclusions: Poorer oral hygiene, older age, current smoking, and diabetes mellitus were associated with a greater number of missing teeth after adjustment for the other measured covariates. Because current oral hygiene and cumulative tooth loss were assessed at the same visit, temporal and causal relationships cannot be established. Furthermore, multimorbidity and polypharmacy were not systematically assessed, and their potential contribution to the observed associations could not be evaluated.

## 1. Introduction

Tooth loss remains a major global public health concern and represents the cumulative outcome of oral diseases, primarily dental caries and periodontal disease [1]. Despite substantial improvements in preventive dentistry, complete or partial tooth loss continues to affect large segments of the adult population worldwide and is associated with impaired mastication, reduced quality of life, nutritional deficiencies and adverse systemic health outcomes. Recent global estimates indicate that severe tooth loss and periodontal disease contribute substantially to years lived with disability, underscoring the need to better understand modifiable factors associated with tooth retention [2,3].

Oral hygiene plays a central role in the prevention of dental caries and periodontal disease by limiting dental plaque accumulation and controlling gingival inflammation. Poor oral hygiene has been consistently linked to increased prevalence and severity of periodontal disease, which is a leading cause of tooth loss in adults. Epidemiologic studies have demonstrated associations between inadequate oral hygiene practices, periodontal tissue destruction and subsequent tooth extraction. However, oral health does not exist in isolation and tooth loss is increasingly recognized as a condition influenced by systemic health and shared behavioral risk factors [4,5].

Major cardiovascular risk factors, including smoking, diabetes mellitus, obesity, hypertension and dyslipidemia are associated with chronic inflammatory and metabolic disturbances that may also affect periodontal tissues [6]. Among these factors, smoking and diabetes mellitus are well-established contributors to periodontal disease progression and tooth loss. In contrast, the adjusted associations of other cardiovascular risk factors, such as hypertension, obesity and dyslipidemia, remain less clearly defined, with previous studies reporting inconsistent findings. These inconsistent findings may reflect differences in study design, population characteristics, outcome definitions and the extent of confounder control [7,8,9]. In addition to individual cardiovascular risk factors, multimorbidity and polypharmacy may represent important considerations when examining oral health in adult clinical populations. Multimorbidity, generally defined as the coexistence of two or more chronic conditions, may be associated with greater cumulative disease burden, functional limitations, and challenges in maintaining oral hygiene [10]. Polypharmacy, commonly defined as the concurrent use of five or more medications, may also influence oral health through medication-related adverse effects, including xerostomia, which can contribute to oral disease [11,12]. However, these factors require specific assessment and should not be inferred solely from the presence of individual cardiovascular risk factors. Their potential role provides additional context for interpreting associations between systemic health and tooth loss.

The relationship between periodontal disease and atherosclerotic cardiovascular disease has been extensively investigated, with growing evidence supporting shared inflammatory and immunological pathways. Chronic periodontal infection may contribute to systemic inflammation and endothelial dysfunction, while cardiometabolic disorders may exacerbate periodontal breakdown. Nevertheless, many studies examining oral–systemic links have focused on periodontal indices or cardiovascular outcomes, rather than on tooth loss as a clinically meaningful and cumulative endpoint [13,14,15]. Examining the number of missing teeth in relation to current oral hygiene and systemic risk factors may therefore provide additional information regarding the clinical and behavioral characteristics associated with tooth retention. However, fewer studies have evaluated oral hygiene and several commonly recorded cardiovascular risk factors simultaneously in relation to the number of missing teeth within the same ambulatory adult population [16,17,18].

Although associations of tooth loss with oral hygiene, smoking, diabetes mellitus, and age have been reported previously, the present study was not designed to establish these factors as novel causes of tooth loss. Rather, it evaluates their simultaneous cross-sectional associations with the number of missing natural teeth within the same ambulatory clinical population while accounting for several commonly recorded cardiovascular risk factors. The study additionally examines whether the association between clinically assessed oral hygiene status and tooth loss shows a similar pattern across sex and age subgroups. These analyses provide clinical-sample evidence regarding the relative pattern of measured associations while recognizing that temporal and causal relationships cannot be established.

## 2. Material and Methods

### 2.1. Study Design and Population

This cross-sectional observational study was conducted among ambulatory adults evaluated between April and August 2026 at the Clinical County Emergency Hospital Oradea and four affiliated secondary medical centers. A total of 1031 adult participants were included in the study. Participants initially underwent a clinical dental examination, after which they were referred to the Outpatient Cardiology Department of the Clinical County Emergency Hospital Oradea for cardiovascular evaluation and were included in the study. The cardiovascular assessment included a systematic evaluation of established cardiovascular risk factors and other clinically relevant parameters in accordance with the study protocol. Demographic, dental, and cardiovascular data were collected during these evaluations and subsequently integrated for analysis.

The final analytical population comprised 1031 participants with complete data for the variables required for the analysis. Detailed screening logs documenting the total number of individuals initially assessed for eligibility, the number excluded at each stage and for each specific reason, and the number recruited from each individual clinical setting were not retained in a form that permitted reliable retrospective reconstruction. Consequently, these numbers are not reported to avoid introducing potentially inaccurate information.

Data collection was conducted across the main hospital and affiliated clinical settings during the five-month study period. The dental assessment used for the present analysis was focused on the study variables, principally the clinical count of missing natural teeth and the qualitative assessment of oral hygiene status, and was not intended to constitute a comprehensive periodontal examination. Examination duration per participant and center-specific patient throughput were not systematically recorded and therefore cannot be reported retrospectively.

The study was conducted in accordance with the principles of the Declaration of Helsinki and approved by the Ethics Committee of Clinical County Emergency Hospital Bihor (protocol number 13257/23 April 2026) for studies involving humans. Clinical data were anonymized prior to analysis. All patients had provided informed consent for the use of anonymized clinical data for research purposes.

### 2.2. Inclusion and Exclusion Criteria

Inclusion criteria were: age ≥18 years and availability of complete data on the number of missing teeth, oral hygiene status, age, sex, and cardiovascular risk factors (hypertension, obesity, dyslipidemia, smoking status, and diabetes mellitus).

Exclusion criteria were: congenital dental agenesis; history of maxillofacial trauma or surgery resulting in non-disease-related tooth loss; current orthodontic treatment affecting tooth count; and incomplete or missing oral examination data.

Completely edentulous participants were not excluded. Because third molars were excluded from the assessment, the number of missing natural teeth ranged from 0 to 28; a value of 28 therefore represented complete absence of the 28 assessed natural teeth. These participants were retained because complete tooth loss represents the upper end of the cumulative tooth-loss outcome evaluated in this study.

### 2.3. Assessment of Missing Teeth

Prior to cardiovascular evaluation, oral examinations were performed in hospital-affiliated dental clinics. Dental screening was conducted either by a senior dental student under the direct supervision of an experienced dental medicine professional or by the dental medicine professional. Before patient screening began, the examination procedure and clinical assessment approach were reviewed with the examiners, including the identification and counting of present and missing natural teeth, exclusion of third molars from the tooth count, and the clinical features considered in the qualitative oral-hygiene assessment. Examinations performed by the senior dental student were conducted under direct clinical supervision. This procedure represented pre-study standardization and supervision rather than a formal examiner-calibration study.

The primary dental outcome was the number of missing natural teeth determined during the clinical oral examination. Third molars were excluded, yielding a possible range of 0 to 28 missing teeth. Participants with 28 missing teeth were retained and represented the maximum value of the outcome.

The historical cause of loss of each individual tooth was not systematically verified using previous dental records, historical radiographs, or a standardized tooth-specific etiological assessment. Therefore, the analysis was based on the number of missing teeth rather than on cause-specific tooth loss, and missing teeth were not attributed analytically to periodontitis, dental caries, or another specific pathological mechanism.

A formal intra- or inter-examiner reliability study involving duplicate independent examinations and calculation of agreement coefficients was not performed. Examiner-specific logs permitting reliable retrospective determination of the number of examinations performed by each examiner were also not available. Examiner consistency was addressed through review of the examination approach before screening and direct clinical supervision of examinations performed by the senior dental student.

### 2.4. Assessment of Oral Hygiene Status

Oral hygiene status was recorded during the clinical dental examination and categorized as good, fair, or poor according to the qualitative clinical assessment used at the time of screening.

Classification was based on visual inspection of dental plaque accumulation, gingival condition and presence of calculus.

• Good oral hygiene was defined as minimal or no visible plaque, absence of calculus and healthy gingival appearance.
• Fair oral hygiene was defined as moderate plaque accumulation and/or localized gingival inflammation.
• Poor oral hygiene was defined as extensive plaque accumulation, generalized calculus deposits and signs of gingival inflammation.

A validated quantitative plaque or oral hygiene index was not used, and the original screening records did not contain quantitative thresholds or cut-off values that would permit retrospective reconstruction of standardized criteria for each category. Similarly, a formal decision rule for cases with mixed clinical findings was not documented. Accordingly, no post hoc quantitative criteria were created for the present analysis.

A comprehensive periodontal examination was not part of the study protocol. Probing pocket depth, clinical attachment level, bleeding on probing, gingival recession, tooth mobility, periodontal staging or grading, and radiographic alveolar bone loss were not systematically recorded. Consequently, periodontal disease status and severity could not be included as measured covariates, and the present analysis cannot determine whether individual missing teeth were attributable to periodontitis.

### 2.5. Assessment of Cardiovascular Risk Factors

The following cardiovascular risk factors were recorded by cardiologists and coded as binary variables (present/absent):

• Hypertension: documented diagnosis or current antihypertensive treatment.
• Obesity: body mass index (BMI) ≥ 30 kg/m^2^.
• Dyslipidemia: documented diagnosis or current lipid-lowering therapy.
• Smoking status: current smoking at the time of assessment.
• Diabetes mellitus: documented diagnosis or current use of glucose-lowering medication.

Age (years) and sex (male/female) were also recorded.

The assessment focused on the predefined cardiovascular risk factors included in the study protocol. Although several chronic conditions were recorded individually, a comprehensive assessment of multimorbidity was not performed, and a standardized multimorbidity index was not calculated. Furthermore, detailed information regarding the total number, classes, and duration of medications used by each participant was not systematically collected. Consequently, polypharmacy could not be defined or evaluated as a separate variable in the present analysis.

### 2.6. Statistical Analysis

Continuous variables were summarized as mean ± standard deviation and, where appropriate, median and interquartile range; categorical variables were presented as frequencies and percentages. Differences in the distribution of missing teeth across oral hygiene categories were evaluated using the Kruskal–Wallis test. Comparisons between participants with and without each cardiovascular risk factor were performed using two-sided Mann–Whitney U tests. These analyses were considered unadjusted comparisons.

The number of missing teeth was analyzed as a count outcome. Negative binomial regression was used as the primary multivariable model, with results reported as incidence rate ratios (IRRs) and 95% confidence intervals (CIs). Dispersion was assessed using the corresponding Poisson model. The Pearson chi-square/df ratio was 1.09 and the deviance/df ratio was 1.16, indicating a modest departure from equidispersion. The negative binomial model showed a slightly lower Akaike information criterion than the Poisson model (AIC 5393.6 versus 5394.7) and was retained as the primary count model. The estimated negative-binomial dispersion parameter was α = 0.0069.

The adjusted model included oral hygiene status, age, sex, hypertension, obesity, dyslipidemia, current smoking, and diabetes mellitus. Good oral hygiene and absence of each binary risk factor served as reference categories. Multicollinearity among the predictors was assessed using variance inflation factors (VIFs) and tolerance values. The functional form of age was additionally examined by comparing the primary model containing age as a linear continuous term with a sensitivity model including both linear and quadratic age terms. Oral hygiene status was modeled using separate indicator variables for the fair and poor categories, with good oral hygiene as the reference category; therefore, no linear relationship across the three oral-hygiene categories was assumed. Pairwise correlations among model predictors were also examined as supplementary diagnostics.

As a supportive analysis, multivariable linear regression treating the number of missing teeth as a continuous outcome was performed to assess consistency in the direction of associations. Results are presented as adjusted regression coefficients with 95% CIs.

### 2.7. Supplementary Analyses

Exploratory subgroup analyses were performed by sex and by age (<65 and ≥65 years). Effect modification by sex was evaluated by adding oral hygiene × sex interaction terms to the adjusted count model and applying a joint Wald test. For subgroup negative binomial models, the dispersion parameter was fixed at α = 0.0069, estimated from the full-sample model.

As an additional model-adequacy analysis, a sensitivity model including a quadratic age term was fitted to evaluate potential nonlinearity in the association between age and the expected number of missing teeth. For this sensitivity analysis, the negative-binomial dispersion parameter was fixed at the value estimated from the primary full-sample model (α = 0.0069), allowing the functional form of age to be compared while retaining the same dispersion specification.

Statistical analyses were performed using Python version 3.13.5, with statsmodels version 0.14.6 for regression analyses and SciPy version 1.17.0 for nonparametric tests. All tests were two-sided, and *p* < 0.05 was considered statistically significant.

## 3. Results

### 3.1. Study Population

The analytical sample comprised 1031 adult ambulatory patients, including 579 women (56.2%) and 452 men (43.8%). Mean age was 54.6 ± 11.8 years (median 55; interquartile range 47–62; range 18–90 years). Oral hygiene was classified as good in 236 participants (22.9%), fair in 437 (42.4%), and poor in 358 (34.7%). Hypertension was present in 346 participants (33.6%), obesity in 260 (25.2%), dyslipidemia in 225 (21.8%), current smoking in 298 (28.9%), and diabetes mellitus in 112 (10.9%). Multimorbidity and polypharmacy were not evaluated as separate study variables; therefore, no descriptive or inferential results are reported for these characteristics.

The mean number of missing natural teeth was 11.01 ± 5.70 (median 11; interquartile range 7–15; range 0–28). Eleven participants (1.07%) had 28 missing teeth and were retained in the analytical population. All participants had complete data for the variables included in the analysis.

### 3.2. Descriptive Analysis

#### 3.2.1. Missing Teeth by Oral Hygiene Status

The number of missing teeth differed markedly according to oral hygiene status (Table 1). Participants with good oral hygiene had the lowest mean number of missing teeth, whereas those with fair and poor oral hygiene exhibited progressively higher tooth loss. Mean numbers of missing teeth were 7.74 ± 4.83 in the good oral hygiene group, 10.02 ± 4.73 in the fair group and 14.36 ± 5.62 in the poor group, indicating a clear stepwise gradient across categories. The distribution of missing teeth differed significantly across oral hygiene categories (Kruskal–Wallis *p* < 0.001; Table 1). These findings represent unadjusted comparisons; adjusted associations are presented in the multivariable regression analysis.

Post hoc pairwise comparisons using Mann–Whitney U tests with Bonferroni correction showed significant differences between good and fair, good and poor, and fair and poor oral hygiene categories (all adjusted *p* < 0.001).

#### 3.2.2. Missing Teeth by Cardiovascular Risk Factors

In unadjusted analyses, the distribution of missing teeth differed significantly according to hypertension, obesity, dyslipidemia, current smoking, and diabetes mellitus status (all *p* < 0.001; Table 2). Participants with each of these risk factors had higher mean numbers of missing teeth than participants without the corresponding risk factor. The largest unadjusted difference was observed for diabetes mellitus. These descriptive associations should be distinguished from the multivariable results, in which hypertension, obesity, and dyslipidemia were not statistically significant in the fully adjusted model.

#### 3.2.3. Multivariable Negative Binomial Regression (Primary Analysis)

In the multivariable negative binomial regression model (Table 3), oral hygiene status was significantly associated with the number of missing teeth after adjustment for age, sex, hypertension, obesity, dyslipidemia, current smoking, and diabetes mellitus. Compared with participants with good oral hygiene, those with fair oral hygiene had a higher expected number of missing teeth (IRR 1.179, 95% CI 1.114–1.248, *p* < 0.001), as did those with poor oral hygiene (IRR 1.490, 95% CI 1.406–1.579, *p* < 0.001).

Increasing age was associated with a higher expected number of missing teeth (IRR 1.031 per year, 95% CI 1.029–1.033, *p* < 0.001). Current smoking (IRR 1.147, 95% CI 1.099–1.198, *p* < 0.001) and diabetes mellitus (IRR 1.118, 95% CI 1.056–1.182, *p* < 0.001) were also associated with a higher expected number of missing teeth after adjustment for the other measured covariates. Sex (*p* = 0.512), hypertension (*p* = 0.903), obesity (*p* = 0.109), and dyslipidemia (*p* = 0.865) were not statistically significant in the fully adjusted model.

#### 3.2.4. Multivariable Linear Regression (Supportive Analysis)

The supportive multivariable linear regression analysis showed a pattern consistent with the primary count model (Table 4). Compared with good oral hygiene, fair oral hygiene was associated with 1.28 additional missing teeth (β = 1.276, 95% CI 0.762–1.790, *p* < 0.001), while poor oral hygiene was associated with 4.28 additional missing teeth (β = 4.279, 95% CI 3.719–4.840, *p* < 0.001), after adjustment for the other variables in the model. Each additional year of age was associated with approximately 0.33 additional missing teeth (β = 0.328, 95% CI 0.310–0.346, *p* < 0.001). Current smoking (β = 1.292, 95% CI 0.838–1.745, *p* < 0.001) and diabetes mellitus (β = 1.695, 95% CI 1.046–2.344, *p* < 0.001) were also associated with a greater number of missing teeth. Sex, hypertension, obesity, and dyslipidemia were not statistically significant in this model.

**Figure 1:**
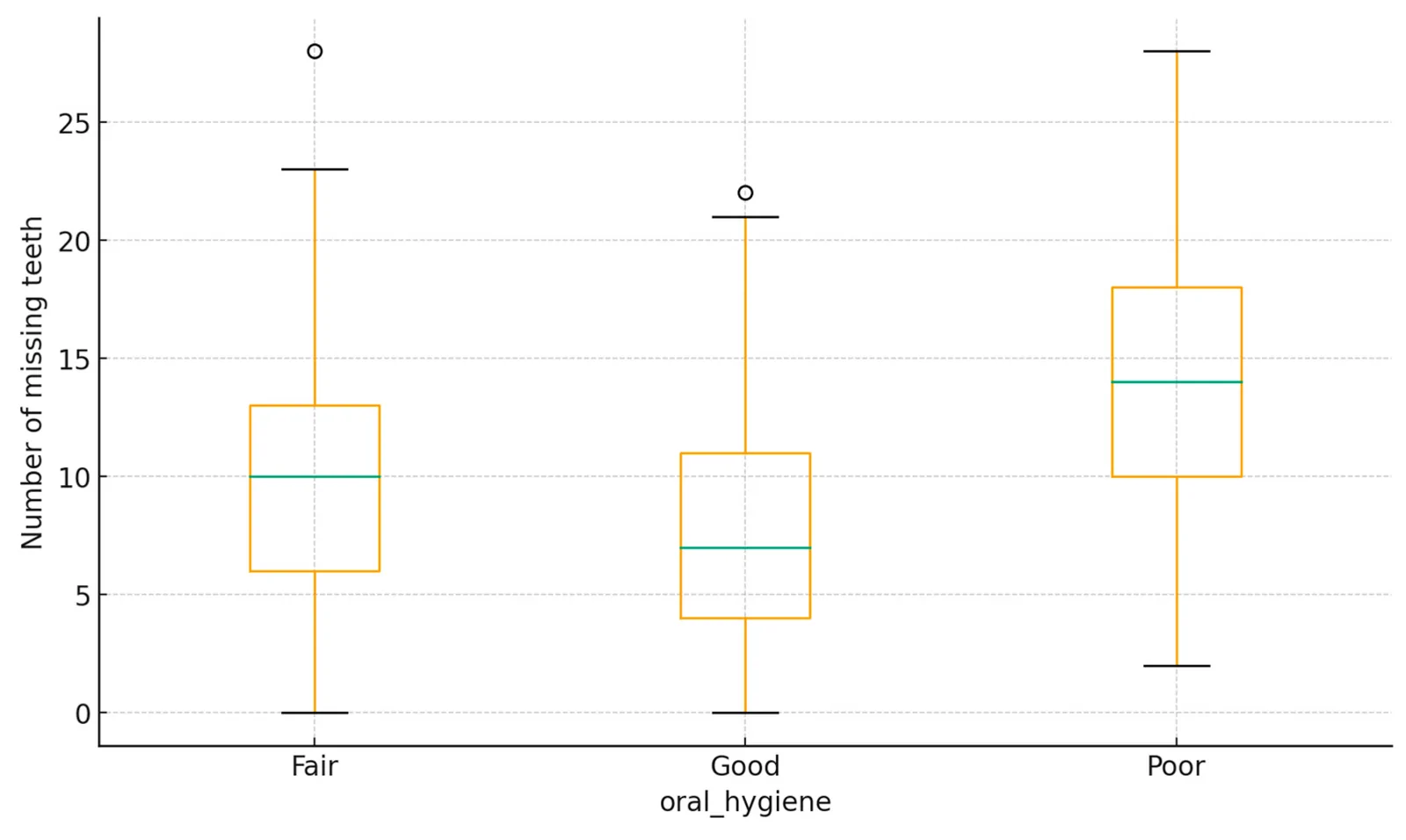
Distribution of missing natural teeth according to oral hygiene status.

Figure 1 illustrates the distribution of missing natural teeth across oral hygiene categories.

**Figure 2:**
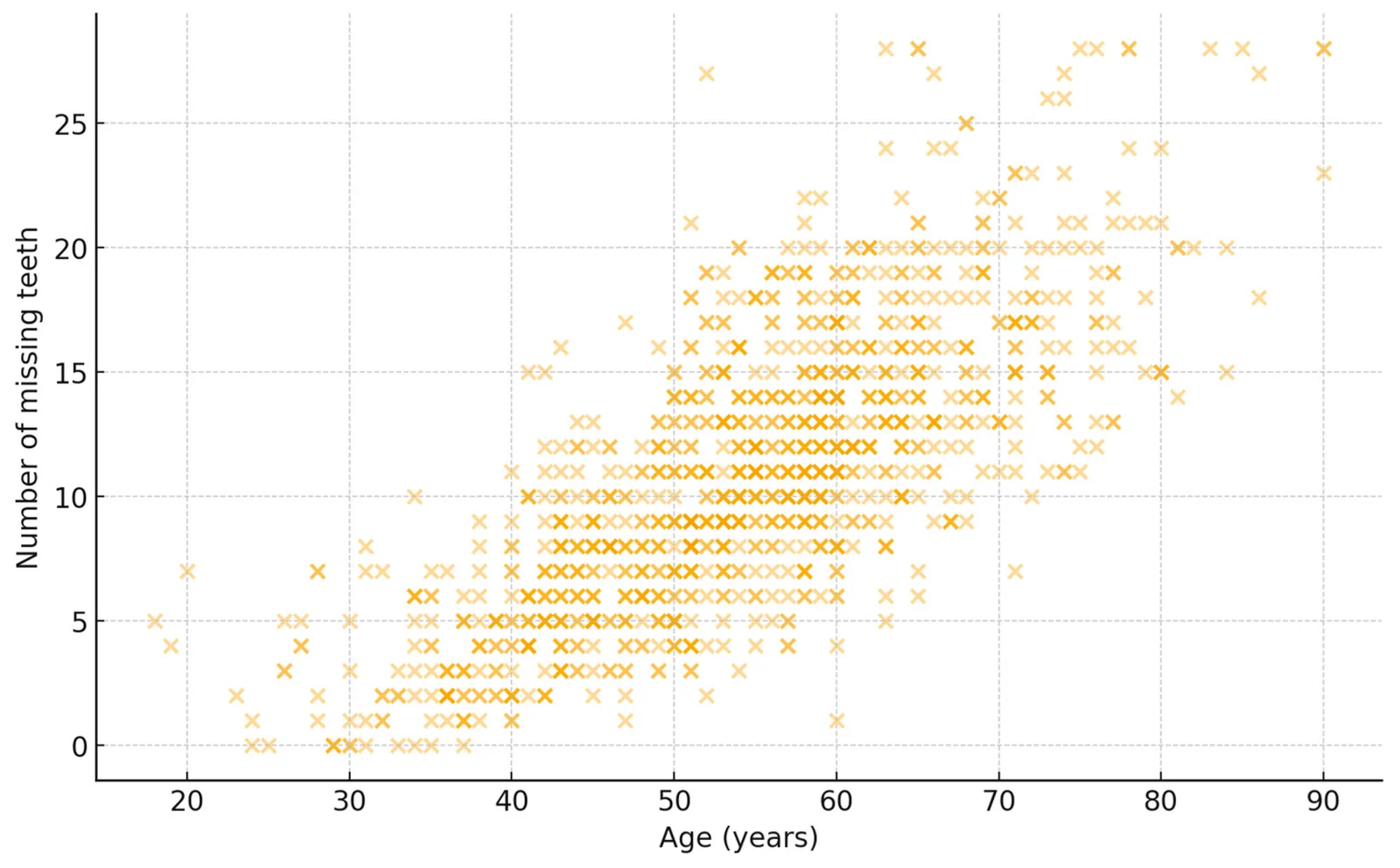
Relationship between age and number of missing natural teeth.

Figure 2 illustrates the relationship between age and the number of missing natural teeth. Each point represents one participant. The solid line represents the fitted linear regression line.

#### 3.2.5. Stratified and Interaction Analyses

Sex-stratified analyses showed consistent associations between oral hygiene status and the number of missing teeth. Among women (n = 579), the adjusted IRRs were 1.184 (95% CI 1.094–1.282) for fair versus good oral hygiene and 1.485 (95% CI 1.370–1.610) for poor versus good oral hygiene. Among men (n = 452), the corresponding IRRs were 1.172 (95% CI 1.080–1.272) and 1.497 (95% CI 1.376–1.628), respectively. Formal testing provided no evidence that the association between oral hygiene and tooth loss differed by sex (joint oral hygiene × sex interaction: Wald χ^2^ [2] = 0.780, *p* = 0.677).

In age-stratified analyses, poor versus good oral hygiene remained associated with a higher expected number of missing teeth among participants aged <65 years (n = 834; IRR 1.585, 95% CI 1.481–1.695) and those aged ≥65 years (n = 197; IRR 1.250, 95% CI 1.114–1.402). Fair versus good oral hygiene was associated with tooth loss in the younger group (IRR 1.241, 95% CI 1.162–1.325) but not in the older group (IRR 0.977, 95% CI 0.871–1.096). These subgroup analyses were considered exploratory, particularly because of the smaller size of the ≥65-year subgroup.

Current smoking was associated with a higher expected number of missing teeth in women, men, and participants aged <65 years, but not in the ≥65-year subgroup. Diabetes mellitus was associated with a higher expected number of missing teeth in both sex strata and both age strata. Hypertension, obesity, and dyslipidemia were not statistically significant in any of the stratified models. These subgroup analyses were exploratory, and differences in statistical significance between strata should not be interpreted as evidence of effect modification in the absence of a corresponding formal interaction test.

The complete stratified models are presented in Supplementary Table S1.

#### 3.2.6. Model Diagnostics and Sensitivity Analysis

No evidence of problematic multicollinearity was identified among the variables included in the adjusted model. Variance inflation factors ranged from 1.01 to 1.84, with all tolerance values > 0.54. Pairwise correlations among the predictors were generally modest; the correlation between the indicator variables for fair and poor oral hygiene reflected their mutually exclusive coding within the same categorical variable.

A sensitivity analysis indicated evidence of nonlinearity in the association between age and the expected number of missing teeth. With the negative-binomial dispersion parameter fixed at α = 0.0069 in both sensitivity models, inclusion of a quadratic age term improved model fit compared with the model containing age as a linear term alone (AIC 5290.2 versus 5391.6; likelihood-ratio *p* < 0.001). Importantly, allowing for nonlinearity in age did not materially alter the principal associations. Fair oral hygiene (IRR 1.178, 95% CI 1.113–1.246; *p* < 0.001) and poor oral hygiene (IRR 1.501, 95% CI 1.416–1.591; *p* < 0.001) remained associated with higher expected numbers of missing teeth compared with good oral hygiene. Current smoking (IRR 1.124, 95% CI 1.077–1.174; *p* < 0.001) and diabetes mellitus (IRR 1.131, 95% CI 1.069–1.196; *p* < 0.001) also remained statistically significant. Thus, the principal findings were robust to allowing a nonlinear functional form for age.

## 4. Discussion

### 4.1. Principal Findings

The principal finding of this study was a graded cross-sectional association between clinically assessed oral hygiene status and cumulative tooth loss. After adjustment for age, sex, hypertension, obesity, dyslipidemia, current smoking, and diabetes mellitus, both fair and poor oral hygiene were associated with a higher expected number of missing teeth compared with good oral hygiene, with the larger estimate observed for poor oral hygiene. Older age, current smoking, and diabetes mellitus were also associated with greater tooth loss.

These findings should be interpreted as cross-sectional associations rather than causal effects. In particular, current oral hygiene status may not reflect oral hygiene during the period in which tooth loss occurred, and extensive tooth loss may itself influence the clinical assessment of oral hygiene.

### 4.2. Oral Hygiene and Tooth Loss

A graded association was observed across oral hygiene categories, with higher expected numbers of missing teeth among participants classified as having fair or poor oral hygiene compared with those classified as having good oral hygiene. This pattern is consistent with previous literature describing relationships among oral hygiene, plaque accumulation, oral disease, and tooth retention. However, the present study does not establish the biological pathway responsible for the observed association.

Periodontal inflammation and tissue destruction represent one biologically plausible pathway linking long-term plaque accumulation with tooth loss, as described in the previous literature [1,19]. This mechanism was not directly evaluated in the present study because standardized periodontal parameters were not collected. Furthermore, the historical cause of individual tooth loss could not be reliably distinguished between periodontal disease, dental caries, and other causes.

Because oral hygiene status and cumulative tooth loss were assessed at the same visit, temporality cannot be established. Current oral hygiene may not reflect oral hygiene practices during the years in which teeth were lost, and extensive tooth loss may itself influence the clinical assessment of oral hygiene. The observed association should therefore not be interpreted as demonstrating that current oral hygiene status caused the recorded tooth loss.

### 4.3. Age and Tooth Loss

Increasing age was associated with a higher expected number of missing teeth after adjustment for the other measured covariates. Sensitivity analysis indicated that the age-tooth loss relationship was not completely linear; however, allowing for nonlinearity did not materially alter the principal associations. This finding is consistent with the cumulative nature of tooth loss over the life course, during which individuals may experience repeated exposure to dental caries, periodontal disease, dental treatment, and historical differences in preventive care. Age should therefore be interpreted as an important correlate of accumulated tooth loss rather than as a directly modifiable causal exposure. The association between oral hygiene category and missing teeth remained statistically significant after adjustment for age, although residual confounding by historical oral-health behaviors and other age-related factors cannot be excluded.

### 4.4. Smoking, Diabetes Mellitus and Tooth Loss

Current smoking and diabetes mellitus were associated with a higher expected number of missing teeth after adjustment for the other measured covariates. The association with current smoking is consistent with previous literature describing greater periodontal destruction and more extensive tooth loss among smokers [8,20]. Potential mechanisms described in the literature include altered host inflammatory and immune responses and impaired periodontal healing; however, these mechanisms were not directly evaluated in the present study.

Diabetes mellitus was also associated with a higher expected number of missing teeth. Previous studies have reported associations between diabetes, periodontal disease, and tooth loss [7,21] with hyperglycemia, altered host responses, and microvascular changes proposed as potential mechanisms. Because diabetes duration, type, and glycemic control were not available and periodontal status was not systematically measured, the present results should be interpreted as an adjusted cross-sectional association rather than evidence of a specific biological pathway or temporal effect.

### 4.5. Other Cardiovascular Risk Factors

Although participants with hypertension, obesity, and dyslipidemia had higher observed numbers of missing teeth in the unadjusted comparisons, the adjusted estimates for these variables were not statistically significant in the multivariable model. These findings should not be interpreted as evidence that these conditions have no relationship with oral health, nor do they demonstrate that the unadjusted associations were explained by confounding. Rather, the present analysis did not identify statistically significant adjusted associations for hypertension, obesity, or dyslipidemia after accounting for the measured covariates. Residual confounding, exposure misclassification, and the binary characterization of these conditions should also be considered.

### 4.6. Oral Health and Systemic Disease Considerations

Previous research has described shared behavioral and inflammatory pathways between oral disease and systemic cardiometabolic conditions [22]. Periodontal disease has been associated with systemic inflammation and cardiovascular outcomes, while several systemic conditions may influence periodontal health. However, periodontal status and cardiovascular outcomes were not evaluated as outcomes in the present analysis. Accordingly, these mechanisms provide biological context for the observed associations.

### 4.7. Comparison with Other Studies

The graded association observed between oral-hygiene status and the number of missing teeth is broadly consistent with previous evidence linking oral-health behaviors and disease burden with long-term tooth retention. Longitudinal evidence has identified oral-hygiene practices and smoking among factors associated with subsequent tooth loss [23]. Dental caries and periodontal disease may also coexist; Mattila et al. reported greater occurrence of dental caries among individuals with periodontal disease, particularly those with more severe periodontal involvement [24]. This coexistence emphasizes that cumulative tooth loss cannot be attributed to a single oral-disease mechanism without tooth-specific etiological information.

The observed association between current smoking and a greater number of missing teeth is consistent with previous literature describing greater periodontal destruction and tooth loss among smokers [20]. Similarly, the association with diabetes mellitus is consistent with systematic evidence linking type 2 diabetes with tooth loss [7]. Altered inflammatory responses, impaired host defense, and diabetes-related microvascular changes have been proposed as mechanisms connecting diabetes with periodontal breakdown [21]; however, these mechanisms were not directly evaluated in the present study.

The oral–systemic literature provides additional context for these findings. Extensive tooth loss has been associated with cardiovascular mortality [25], while oral-hygiene practices, including tooth brushing and interdental cleaning, have been associated with cardiometabolic outcomes in systematic reviews and meta-analyses [26,27]. Periodontitis has also been associated with incident atherosclerotic cardiovascular disease [28] and cerebrovascular events [29]. These observations support shared oral–systemic risk pathways but should not be interpreted as evidence that such mechanisms were demonstrated in our study, in which periodontal status and cardiovascular outcomes were not directly evaluated.

Hypertension, obesity, and dyslipidemia were not statistically significant in our fully adjusted model. Previous literature concerning cardiometabolic factors, periodontal disease, and tooth loss has produced heterogeneous findings [5,9,30], and differences in population characteristics, exposure definitions, socioeconomic factors, dental-care access, and confounder adjustment may contribute to this variability. Recent cross-sectional evidence has also described associations between oral-health status, tooth loss, and cardiovascular risk profiles [31].

Taken together, our findings are consistent with previous evidence linking tooth loss with oral hygiene, smoking, and diabetes, while also illustrating the complexity of relationships between cumulative oral-health outcomes and cardiovascular risk factors. The present results should therefore be viewed as additional cross-sectional evidence from an ambulatory clinical population rather than as evidence of new causal relationships.

### 4.8. Strengths and Limitations

The study has several strengths. It included a relatively large ambulatory clinical sample (n = 1031) with complete data for all variables included in the primary analysis. Tooth presence or absence and oral-hygiene status were assessed clinically rather than by self-report. The number of missing teeth was analyzed as a count outcome using negative binomial regression, with a supportive linear regression analysis showing a broadly consistent pattern of associations. Additional model diagnostics showed no evidence of problematic multicollinearity, and sensitivity analysis allowing a nonlinear association with age did not materially alter the principal findings. Exploratory sex- and age-stratified analyses and formal testing of the oral hygiene × sex interaction provided further information regarding the consistency of the observed associations across clinical subgroups.

Several limitations should be considered. First, the cross-sectional design precludes establishing temporality or causality. Tooth loss is a cumulative outcome reflecting long-term oral and systemic exposures, whereas oral hygiene and cardiovascular risk factors were assessed at a single time point. Current oral hygiene may therefore not reflect historical behaviors during the period in which teeth were lost, and extensive tooth loss may itself influence the clinical assessment of oral hygiene.

Second, oral and periodontal characterization was limited. Oral hygiene was classified qualitatively as good, fair, or poor without a validated quantitative index, retrospectively verifiable cut-off values, or formal intra- and inter-examiner reliability coefficients. Comprehensive periodontal measurements, including probing depth, clinical attachment loss, and tooth mobility, were not systematically recorded; consequently, residual confounding by unmeasured periodontal disease cannot be excluded. In addition, the historical cause and timing of individual tooth loss were not systematically verified, preventing distinction between tooth loss attributable to caries, periodontitis, or other causes.

Third, several exposures and potential confounders were incompletely characterized or unavailable. Smoking was recorded only as current smoking, without information on former smoking, intensity, duration, or pack-years. Diabetes mellitus was recorded without systematic information on type, duration, HbA1c, or other measures of glycemic control. Hypertension and dyslipidemia did not distinguish controlled from uncontrolled disease, and obesity was defined using BMI without measures of central adiposity. Socioeconomic position, education, income, access to dental care, preventive dental attendance, dietary factors, and use of fluoride-containing oral-health products were not available. Residual confounding by these unmeasured or incompletely characterized factors therefore cannot be excluded. An additional limitation is the absence of a comprehensive assessment of multimorbidity and polypharmacy. Although individual cardiovascular risk factors were recorded, the overall burden of coexisting chronic conditions was not systematically quantified. Similarly, detailed medication-use data were unavailable, preventing an assessment of polypharmacy and potentially relevant medication-related effects on oral health, such as xerostomia. Both multimorbidity and polypharmacy may be associated with oral-health outcomes and could contribute to residual confounding. Consequently, the present findings should be interpreted in the context of these unmeasured factors, and future studies should incorporate standardized assessments of chronic disease burden and medication exposure.

Fourth, age was entered as a linear continuous term in the primary model for ease of interpretation, although sensitivity analysis indicated a nonlinear age–tooth-loss relationship. Importantly, the principal associations with oral hygiene, current smoking, and diabetes mellitus were materially unchanged when a quadratic age term was included.

Fifth, the relatively large number of participants evaluated during the five-month recruitment period should be considered in relation to the focused scope of the dental assessment, which recorded tooth presence or absence and qualitative oral-hygiene status rather than providing comprehensive periodontal charting. Examination duration, center-specific throughput, and detailed historical recruitment-flow data, including the numbers screened and excluded at each stage and the distribution across recruitment settings, were not available for reliable retrospective reconstruction. Selection related to the recruitment process therefore cannot be fully assessed.

Finally, participants were recruited through hospital-affiliated ambulatory settings with subsequent cardiovascular evaluation and should not be considered representative of the general adult population. The prevalence of cardiovascular risk factors observed in this sample should therefore not be extrapolated to the general population, and the magnitude of the observed associations may differ in community-based settings. Confirmation in longitudinal, population-based cohorts using validated oral-hygiene indices, standardized periodontal measurements, and more comprehensive assessment of behavioral, metabolic, socioeconomic, and dental-care factors is warranted.

### 4.9. Clinical and Public Health Implications

The findings of the present study should be interpreted within the limitations of its cross-sectional design. Although poorer oral hygiene, current smoking, diabetes mellitus, and older age were associated with a greater number of missing teeth, these associations do not establish temporal or causal relationships. Consequently, these data do not allow us to recommend changes in clinical practice based on the present findings alone. Instead, they emphasize the need for further longitudinal studies to clarify the temporal relationships between oral hygiene, cardiovascular risk factors, and tooth loss. Future investigations should also incorporate comprehensive assessments of multimorbidity, polypharmacy, periodontal status, and other potential confounding factors to better characterize the complex relationships between oral and systemic health.

## 5. Conclusions

In this cross-sectional study of adult ambulatory patients, poorer clinically assessed oral hygiene, older age, current smoking, and diabetes mellitus were associated with a greater number of missing teeth after adjustment for the other measured covariates. Hypertension, obesity, and dyslipidemia were not statistically significant in the fully adjusted model. These findings confirm associations previously described in the literature within this specific ambulatory clinical population rather than establishing new causal relationships. Because oral hygiene status and cumulative tooth loss were assessed at the same visit, temporal sequence and causality cannot be determined. The qualitative assessment of oral hygiene, absence of standardized periodontal measurements, incomplete characterization of behavioral and systemic exposures, unmeasured socioeconomic and dental-care factors, and clinical recruitment setting should be considered when interpreting the findings. Furthermore, the absence of systematic information on multimorbidity and polypharmacy limits the ability to account for the potential influence of overall chronic disease burden and medication exposure on the observed associations. Longitudinal population-based studies using validated oral-hygiene indices, standardized periodontal assessments, and more comprehensive confounder measurement are needed to clarify temporal relationships and the magnitude of these associations.

## Figures and Tables

**Table 1:** Number of missing teeth by oral hygiene status.

Oral Hygiene Status	n	Mean	SD	Unadjusted *p*-Value
Good	236	7.74	4.83	<0.001
Fair	437	10.02	4.73	
Poor	358	14.36	5.62	

Note: Values are presented as mean ± standard deviation. The *p*-value represents the overall unadjusted comparison of the distribution of missing teeth across the three oral hygiene categories using the Kruskal–Wallis test (H = 203.86, *p* < 0.001). The *p*-value is descriptive and unadjusted; adjusted associations are presented in the multivariable regression analyses.

**Table 2:** Number of missing teeth by cardiovascular risk factors.

Risk Factor	Status	n	Mean	SD	Unadjusted *p*-Value
Hypertension	Absent	685	10.19	5.48	<0.001
	Present	346	12.63	5.79	
Obesity	Absent	771	10.60	5.59	<0.001
	Present	260	12.22	5.86	
Dyslipidemia	Absent	806	10.58	5.61	<0.001
	Present	225	12.53	5.78	
Current smoking	Absent	733	10.47	5.70	<0.001
	Present	298	12.32	5.50	
Diabetes mellitus	Absent	919	10.51	5.45	<0.001
	Present	112	15.08	6.07	

Note: Values are presented as mean ± standard deviation. *p*-values represent two-sided unadjusted comparisons of the distribution of missing teeth between participants with and without each risk factor, using the Mann–Whitney U test. All five comparisons yielded *p* < 0.001. Risk factors were coded as binary variables (present/absent) based on the clinical definitions specified in the Methods. These *p*-values are descriptive and unadjusted; adjusted associations are presented in the multivariable regression analyses. Statistical significance in an unadjusted comparison does not imply an independent association after multivariable adjustment.

**Table 3:** Multivariable negative binomial regression analysis of factors associated with the number of missing teeth (n = 1031).

Variable	Category	IRR	95% CI	*p*-Value
Oral hygiene	Good	1.00 (reference)	—	—
	Fair	1.179	1.114–1.248	<0.001
	Poor	1.490	1.406–1.579	<0.001
Age	Per year	1.031	1.029–1.033	<0.001
Sex	Female	1.00 (reference)	—	—
	Male	1.013	0.974–1.053	0.512
Hypertension	Absent	1.00 (reference)	—	—
	Present	0.997	0.957–1.040	0.903
Obesity	Absent	1.00 (reference)	—	—
	Present	1.037	0.992–1.083	0.109
Dyslipidemia	Absent	1.00 (reference)	—	—
	Present	1.004	0.959–1.051	0.865
Current smoking	Absent	1.00 (reference)	—	—
	Present	1.147	1.099–1.198	<0.001
Diabetes mellitus	Absent	1.00 (reference)	—	—
	Present	1.118	1.056–1.182	<0.001

Note: IRR = incidence rate ratio; CI = confidence interval. Reference categories are shown explicitly as 1.00 (reference). The model was adjusted simultaneously for oral hygiene status, age, sex, hypertension, obesity, dyslipidemia, current smoking, and diabetes mellitus. IRRs >1 indicate a higher expected number of missing teeth relative to the reference category.

**Table 4:** Multivariable linear regression analysis examining associations with the number of missing natural teeth (n = 1031).

Variable	Category	Adjusted β	95% CI	*p*-Value
Oral hygiene	Good	0 (reference)	—	—
	Fair	+1.28	0.76–1.79	<0.001
	Poor	+4.28	3.72–4.84	<0.001
Age	Per year	+0.33	0.31–0.35	<0.001
Sex	Female	0 (reference)	—	—
	Male	+0.09	−0.30–0.49	0.639
Hypertension	Absent	0 (reference)	—	—
	Present	+0.02	−0.42–0.45	0.946
Obesity	Absent	0 (reference)	—	—
	Present	+0.31	−0.15–0.78	0.184
Dyslipidemia	Absent	0 (reference)	—	—
	Present	+0.05	−0.44–0.54	0.832
Current smoking	Absent	0 (reference)	—	—
	Present	+1.29	0.84–1.75	<0.001
Diabetes mellitus	Absent	0 (reference)	—	—
	Present	+1.69	1.05–2.34	<0.001

Note: β coefficients represent adjusted mean differences in the number of missing teeth. Reference categories are shown explicitly as 0 (reference). Each coefficient is adjusted for all other variables included in the model: oral hygiene status, age, sex, hypertension, obesity, dyslipidemia, current smoking, and diabetes mellitus. Positive β values indicate a higher adjusted mean number of missing teeth relative to the reference category.

## Data Availability

The raw data supporting the conclusions of this article will be made available by the authors on request.

## Supplementary Materials

The following supporting information can be downloaded at: https://www.mdpi.com/article/10.3390/life16101717/s1, Table S1: Adjusted negative binomial regression analyses stratified by sex and age group.

## References

[1] Petersen P.E., Ogawa H. (2012). The Global Burden of Periodontal Disease: Towards Integration with Chronic Disease Prevention and Control. Periodontol. 2000.

[2] Kassebaum N.J., Bernabé E., Dahiya M., Bhandari B., Murray C.J.L., Marcenes W. (2014). Global Burden of Severe Tooth Loss: A Systematic Review and Meta-Analysis. J. Dent. Res..

[3] Emami E., De Souza R.F., Kabawat M., Feine J.S. (2013). The Impact of Edentulism on Oral and General Health. Int. J. Dent..

[4] Denny C., Natarajan S., Jophy J., Kp N., Lewis A.J., Yellapurkar S. (2025). Assessing Socioeconomic Status through Dental and Associated Tissue Characteristics: A Cross-Sectional Study for Human Identification. F1000Research.

[5] Peres M.A., Macpherson L.M.D., Weyant R.J., Daly B., Venturelli R., Mathur M.R., Listl S., Celeste R.K., Guarnizo-Herreño C.C., Kearns C. (2019). Oral Diseases: A Global Public Health Challenge. Lancet.

[6] Herrera D., Molina A., Buhlin K., Klinge B. (2020). Periodontal Diseases and Association with Atherosclerotic Disease. Periodontol. 2000.

[7] Ahmadinia A.R., Rahebi D., Mohammadi M., Ghelichi-Ghojogh M., Jafari A., Esmaielzadeh F., Rajabi A. (2022). Association between Type 2 Diabetes (T2D) and Tooth Loss: A Systematic Review and Meta-Analysis. BMC Endocr. Disord..

[8] Borojevic T. (2012). Smoking and Periodontal Disease. Mater. Sociomed..

[9] Nascimento G.G., Leite F.R.M., Conceição D.A., Ferrúa C.P., Singh A., Demarco F.F. (2016). Is There a Relationship between Obesity and Tooth Loss and Edentulism? A Systematic Review and Meta-analysis. Obes. Rev..

[10] Johnston M.C., Crilly M., Black C., Prescott G.J., Mercer S.W. (2019). Defining and Measuring Multimorbidity: A Systematic Review of Systematic Reviews. Eur. J. Public Health.

[11] Srisanoi K., Maniewicz S., Leles C.R., McKenna G., Müller F., Srinivasan M. (2026). Associations Between Multimorbidity, Polypharmacy and Oral Conditions in Older Adults: A Systematic Review and Meta-Analysis. Gerodontology.

[12] Masnoon N., Shakib S., Kalisch-Ellett L., Caughey G.E. (2017). What Is Polypharmacy? A Systematic Review of Definitions. BMC Geriatr..

[13] Marcaccini A.M., Novaes A.B., Meschiari C.A., Souza S.L., Palioto D.B., Sorgi C.A., Faccioli L.H., Tanus-Santos J.E., Gerlach R.F. (2009). Circulating Matrix Metalloproteinase-8 (MMP-8) and MMP-9 Are Increased in Chronic Periodontal Disease and Decrease after Non-Surgical Periodontal Therapy. Clin. Chim. Acta.

[14] Czerniuk M.R., Surma S., Romańczyk M., Nowak J.M., Wojtowicz A., Filipiak K.J. (2022). Unexpected Relationships: Periodontal Diseases: Atherosclerosis–Plaque Destabilization? From the Teeth to a Coronary Event. Biology.

[15] Cinezan C., Rus C.B., Ilias I.T., Cinezan A. (2025). Dental Disease as a Clinical Marker for Coronary Artery Disease Severity: A Narrative Review of Current Evidence and Mechanisms. Medicina.

[16] Joshy G., Arora M., Korda R.J., Chalmers J., Banks E. (2016). Is Poor Oral Health a Risk Marker for Incident Cardiovascular Disease Hospitalisation and All-Cause Mortality? Findings from 172 630 Participants from the Prospective 45 and Up Study. BMJ Open.

[17] King S., Chow C.K., Eberhard J. (2022). Oral Health and Cardiometabolic Disease: Understanding the Relationship. Intern. Med. J..

[18] Cinezan C., Rus C.B., Cinezan A., Ciavoi G. (2026). Tooth Loss as a Predictor of Coronary Artery Disease Severity in Patients with Acute Myocardial Infarction: A Prospective Cross-Sectional Study. J. Clin. Med..

[19] Degasperi G.R., Etchegaray A., Marcelino L., Sicard A., Villalpando K., Pinheiro S.L. (2018). Periodontal Disease: General Aspects from Biofilm to the Immune Response Driven by Periodontal Pathogens. Adv. Microbiol..

[20] Bergström J. (2004). Tobacco Smoking and Chronic Destructive Periodontal Disease. Odontology.

[21] Chapple I.L.C., Genco R., on behalf of Working Group 2 of the Joint EFP/AAP Workshop (2013). Diabetes and Periodontal Diseases: Consensus Report of the Joint EFP/AAP Workshop on Periodontitis and Systemic Diseases. J. Periodontol..

[22] Leira Y., Rodríguez-Yáñez M., Arias S., López-Dequidt I., Campos F., Sobrino T., D’Aiuto F., Castillo J., Blanco J. (2019). Periodontitis Is Associated with Systemic Inflammation and Vascular Endothelial Dysfunction in Patients with Lacunar Infarct. J. Periodontol..

[23] Chambrone L., Chambrone D., Lima L.A., Chambrone L.A. (2010). Predictors of Tooth Loss during Long-term Periodontal Maintenance: A Systematic Review of Observational Studies. J. Clin. Periodontol..

[24] Mattila P.T., Niskanen M.C., Vehkalahti M.M., Nordblad A., Knuuttila M.L.E. (2010). Prevalence and Simultaneous Occurrence of Periodontitis and Dental Caries. J. Clin. Periodontol..

[25] Aminoshariae A., Nosrat A., Jakovljevic A., Jaćimović J., Narasimhan S., Nagendrababu V. (2024). Tooth Loss Is a Risk Factor for Cardiovascular Disease Mortality: A Systematic Review with Meta-Analyses. J. Endod..

[26] Church L., Franks K., Medara N., Curkovic K., Singh B., Mehta J., Bhatti R., King S. (2024). Impact of Oral Hygiene Practices in Reducing Cardiometabolic Risk, Incidence, and Mortality: A Systematic Review. Int. J. Environ. Res. Public Health.

[27] Liu Y., Wang C., Zhang P., Fu W., Zhang J., Zhang Z., Mao J., Yang Y., Zou L. (2025). Association Between the Frequency of Tooth Brushing and the Risk of Cardiovascular Disease: A Systematic Review and Meta-Analysis. Angiology.

[28] Dietrich T., Sharma P., Walter C., Weston P., Beck J. (2013). The Epidemiological Evidence behind the Association between Periodontitis and Incident Atherosclerotic Cardiovascular Disease. J. Clin. Periodontol..

[29] El Masri J., Al Malak A., El Masri D., Ghazi M., Al Boussi S., El Masri Y., Hassoun M., Tlayss M., Salameh P., Hosseini H. (2024). The Association Between Periodontitis, Gingivitis, Tooth Loss and Stroke: An Umbrella Study with Meta-Analysis. Brain Sci..

[30] Muñoz Aguilera E., Suvan J., Buti J., Czesnikiewicz-Guzik M., Barbosa Ribeiro A., Orlandi M., Guzik T.J., Hingorani A.D., Nart J., D’Aiuto F. (2020). Periodontitis Is Associated with Hypertension: A Systematic Review and Meta-Analysis. Cardiovasc. Res..

[31] Grigore I., Hînganu D., Hînganu M.V., Grigore A.G., Voinescu D.C., Matei M.N., Guțu C., Daniel I.T.F., Amariței O., Ciobotaru O.R. (2026). Oral Health and Gut-Targeted Microbial Marker Changes Associated with Prolonged Hospitalization in Cardiac Patients: An Integrative Risk Analysis. Life.

