# Supplementary material for: Interplay Between Oral Health and Cardiovascular Risk Factors in Tooth Loss: Evidence from a Cross-Sectional Analysis
Source: Life (Basel). 2026 Oct 14;16(10):1717. doi: 10.3390/life16101717 (PMC13706342; doi:10.3390/life16101717)
Supplement: Supplementary file 1 [file life-16-01717-s001.zip › life-4576303-supplementary.pdf]

Supplementary Table S1. Adjusted negative binomial regression analyses stratified by sex and age group

Outcome: number of missing natural teeth (third molars excluded). Results are adjusted incidence rate ratios (IRRs), 95% confidence intervals, and p-values.

Variable	Category / comparison	Women (n=579) IRR (95% CI)	p	Men (n=452) IRR (95% CI)	p	Age <65 y (n=834) IRR (95% CI)	p	Age ≥65 y (n=197) IRR (95% CI)	p
Oral hygiene	Good	1.00 (reference)	—	1.00 (reference)	—	1.00 (reference)	—	1.00 (reference)	—
	Fair	1.184 (1.094–1.282)	<0.001	1.172 (1.080–1.272)	<0.001	1.241 (1.162–1.325)	<0.001	0.977 (0.871–1.096)	0.694
	Poor	1.485 (1.370–1.610)	<0.001	1.497 (1.376–1.628)	<0.001	1.585 (1.481–1.695)	<0.001	1.250 (1.114–1.402)	<0.001
Age	Per 1-year increase	1.030 (1.028–1.033)	<0.001	1.032 (1.030–1.035)	<0.001	1.041 (1.038–1.044)	<0.001	1.013 (1.007–1.020)	<0.001
Sex	Female	Not applicable	—	Not applicable	—	1.00 (reference)	—	1.00 (reference)	—
	Male	Not applicable	—	Not applicable	—	1.005 (0.959–1.053)	0.835	1.011 (0.940–1.088)	0.761
Hypertension	Absent	1.00 (reference)	—	1.00 (reference)	—	1.00 (reference)	—	1.00 (reference)	—
	Present	0.994 (0.940–1.050)	0.825	1.003 (0.942–1.068)	0.924	1.015 (0.965–1.067)	0.572	0.984 (0.914–1.059)	0.664
Obesity	Absent	1.00 (reference)	—	1.00 (reference)	—	1.00 (reference)	—	1.00 (reference)	—
	Present	1.022 (0.964–1.084)	0.462	1.058 (0.990–1.130)	0.096	1.047 (0.993–1.103)	0.090	0.984 (0.908–1.066)	0.695
Dyslipidemia	Absent	1.00 (reference)	—	1.00 (reference)	—	1.00 (reference)	—	1.00 (reference)	—
	Present	0.981 (0.921–1.045)	0.559	1.031 (0.964–1.102)	0.378	0.996 (0.942–1.053)	0.887	1.006 (0.927–1.091)	0.888
Current smoking	Absent	1.00 (reference)	—	1.00 (reference)	—	1.00 (reference)	—	1.00 (reference)	—
	Present	1.125 (1.063–1.191)	<0.001	1.180 (1.105–1.261)	<0.001	1.161 (1.105–1.220)	<0.001	1.004 (0.917–1.100)	0.924
Diabetes mellitus	Absent	1.00 (reference)	—	1.00 (reference)	—	1.00 (reference)	—	1.00 (reference)	—
	Present	1.125 (1.040–1.218)	0.003	1.113 (1.026–1.208)	0.010	1.096 (1.020–1.178)	0.012	1.176 (1.070–1.292)	<0.001

Notes. CI = confidence interval; IRR = incidence rate ratio. Each subgroup model included oral hygiene status, age, hypertension, obesity, dyslipidemia, current smoking, and diabetes mellitus; the age-stratified models additionally included sex. Sex is not applicable as a covariate within the sex-stratified models. Good oral hygiene and absence of each binary cardiovascular risk factor served as reference categories. Continuous age was retained within both age strata (<65 and ≥65 years).

For the subgroup analyses, the negative-binomial dispersion parameter was fixed at $\alpha = 0.0069$, estimated from the full-sample model, to provide stable subgroup estimates. In the full-sample interaction model, there was no evidence that the association between oral hygiene status and missing teeth differed by sex (joint Wald test for oral hygiene × sex interaction: $\chi^2(2) = 0.780$, $p = 0.677$). Individual interaction terms were also not statistically significant (fair hygiene × male: $p = 0.961$; poor hygiene × male: $p = 0.557$).
